# Genetic dissection of QTLs associated with spikelet-related traits and grain size in sorghum

**DOI:** 10.1038/s41598-021-88917-x

**Published:** 2021-04-30

**Authors:** Hideki Takanashi, Mitsutoshi Shichijo, Lisa Sakamoto, Hiromi Kajiya-Kanegae, Hiroyoshi Iwata, Wataru Sakamoto, Nobuhiro Tsutsumi

**Affiliations:** 1 Graduate School of Agricultural and Life Sciences, The University of Tokyo, 1-1-1 Yayoi, Bunkyo-ku, Tokyo, 113-8657 Japan; 2 Institute of Plant Science and Resources, Okayama University, Kurashiki, Okayama 710-0046 Japan; 3 Present Address: Research Center for Agricultural Information Technology, National Agriculture and Food Research Organization, Chiyoda-ku, Tokyo, 100-0013 Japan

**Keywords:** Developmental biology, Genetics, Plant sciences

## Abstract

Although spikelet-related traits such as size of anther, spikelet, style, and stigma are associated with sexual reproduction in grasses, no QTLs have been reported in sorghum. Additionally, there are only a few reports on sorghum QTLs related to grain size, such as grain length, width, and thickness. In this study, we performed QTL analyses of nine spikelet-related traits (length of sessile spikelet, pedicellate spikelet, pedicel, anther, style, and stigma; width of sessile spikelet and stigma; and stigma pigmentation) and six grain-related traits (length, width, thickness, length/width ratio, length/thickness ratio, and width/thickness ratio) using sorghum recombinant inbred lines. We identified 36 and 7 QTLs for spikelet-related traits and grain-related traits, respectively, and found that most sorghum spikelet organ length- and width-related traits were partially controlled by the dwarf genes *Dw1* and *Dw3*. Conversely, we found that these *Dw* genes were not strongly involved in the regulation of grain size. The QTLs identified in this study aid in understanding the genetic basis of spikelet- and grain-related traits in sorghum.

## Introduction

Sorghum [*Sorghum bicolor* (L.) Moench] is the world’s fifth most important C_4_ cereal crop (faostat.fao.org), and is a promising crop with higher stress tolerance than other major cereals^1,2^. Because sorghum is rich in morphological diversity and has a relatively small genome (~ 800 Mb^3,4^) when compared with other C_4_ grasses^5^, it is a suitable genetic model for C_4_ grasses.

Sorghum has a wide range of uses, including the production of grains for food (grain sorghum), forage (forage sorghum), and biomass for bioenergy (biomass/sweet sorghum). Therefore, there is a need to breed sorghum varieties that correspond to their particular use. For example, when considering plant height phenotypes, each favorable trait is used in the breeding process; semi-dwarf phenotypes are important for grain production because they reduce the risk of lodging and make mechanical harvesting more efficient, while non-dwarf phenotypes are important for increased biomass yields. Four major plant height-controlling loci (*Dw1*-*Dw4*) were identified in 1954^6^ and these have mainly been used for sorghum breeding for plant height. The plant height-responsible genes have been identified for three of these four loci (*Dw1*: Sobic.009G230800^7,8^, *Dw2*: Sobic.006G067700^9^, *Dw3*: Sobic.007G163800^10^), which have advanced our knowledge of the molecular mechanisms of plant height regulation in sorghum.

**Figure 1:**
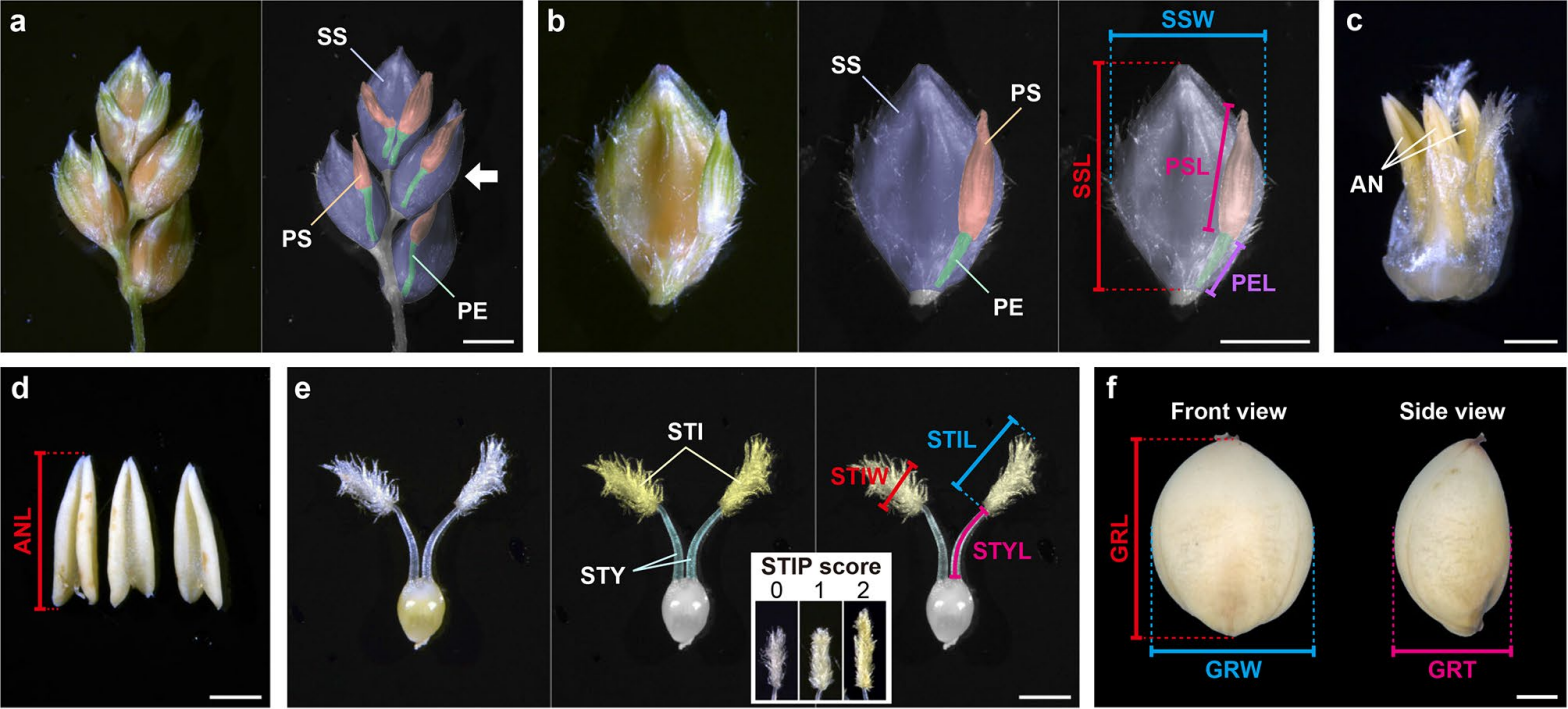
Examples of spikelet-related organs and a mature grain of BTx623. **(a)** The adaxial view of a secondary branch from the BTx623 panicles. The arrow indicates the second-top spikelet pair. **(b)** Enlarged view of the second-top spikelet pair. **(c)** An isolated mature floret from the sessile spikelet. **(d)** Anthers isolated from the floret. **(e)** An isolated pistil from the floret. The inset image shows an example of stigma pigmentation values; we scored white as 0 (BTx623), an intermediate pale yellow color as 1 (RIL065), and yellow as 2 (NOG). **(f)** Front and side views of a mature grain. *SS* sessile spikelet; *PS* pedicellate spikelet; *PE* pedicel; *SSL* sessile spikelet length; *SSW* sessile spikelet width; *PSL* pedicellate spikelet length; *PEL* pedicel length; *AN* anther; *ANL* anther length; *STY* style; *STI* stigma; *STYL* style length; *STIL* stigma length; *STIW* stigma width; *STIP* stigma pigmentation; *GRL* grain length; *GRW* grain width; *GRT* grain thickness. Scale bar = 2 mm in (**a, b**) and 1 mm in **(c–f)**.

Since sorghum grains are important as food, numerous studies have been conducted on traits related to grain weight, and more than 100 quantitative trait loci (QTLs) for grain weight have been identified^11–27^. However, there are only a few reports on sorghum QTLs related to grain size, such as grain length, width, and thickness^28–30^. Sorghum grains, like those of other grasses, are enfolded into floral bracts (glumes, lemmas, and paleas) that form a terminal unit called a spikelet. Sorghum has two types of spikelets: one is a sessile spikelet (SS), and the other is a pedicellate spikelet (PS; Fig. 1a,b). Of these, only SSs are fertile. Recently, it was reported that the PS contributes to seed weight by translocating its photosynthetic products to the SS^31^, suggesting that the PS is not a “useless” organ in sorghum.

Morphologies of spikelet-related organs, such as the glume, lemma, palea, lodicule, anther, and pistil (ovule with style and stigma), are important for sexual reproduction in grasses. For example, it is known that the efficiency of hybrid seed production can be improved by increasing the percentage of exserted stigma, which is closely related to the balance between spikelet size and stigma length in rice^32^. In contrast, cleistogamy, which means autonomous self-pollination caused by the failure of spikelets to open, is a useful genetic tool for the prevention of possible gene transfer in transgenic crops^33^, which maintains the genetic purity of inbreds across generations^34^.

Because of the importance of these morphologies, QTLs and genes that control spikelet-related traits, such as anther length, spikelet length, width, style length, and stigma length, are particularly well studied in rice^32,35–39^. For example, genome-wide association studies for spikelet length and width and style length found that the well-known genes, *GS3* (*GRAIN SIZE 3*, for spikelet length and style length) and *GW5* (*GRAIN WIDTH 5*, for spikelet width) were involved in these traits in rice^36^. *GS3* (*Os03g0407400*) encodes a putative transmembrane protein and has been identified as an evolutionarily important gene that controls grain size and has major effects on rice grain length and weight^40,41^. *GW5*/*qSW5* (*QTL for seed width on chromosome 5*, *Os05g0187500*/*LOC_Os05g09520*) encodes a novel nuclear protein that was identified as a rice domestication-related gene involved in grain width and weight^42,43^. In addition, *SG6* (*SHORT GRAIN 6*, *Os06g0666100*/*LOC_Os06g45540*) controls grain size by regulating spikelet hull (lemma) cell division^37^. *SG6* encodes an uncharacterized plant A/T-rich sequence and zinc-binding protein, and its overexpression resulted in increased plant height and significantly larger and heavier grains. Liu et al. found that *LOC_Os03g14850* was the gene responsible for *qSTL3* (*QTL for stigma length on chromosome 3*), which controls stigma length^32^, while *LOC_Os03g14850* encodes a MADS-box family gene with an M-alpha type-box that affects both stigma length and grain size. Dang et al. reported a novel style length regulator gene, *OsSYL2* (*Os02g0733900*/*LOC_Os02g50110*), which encodes an 80-amino-acid protein with no putative conserved domains^38^. Zhang et al. reported that the grain size (both spikelet length and width) was positively regulated by the Rho-like GTPase *OsRac1* (*Os01g0229400*/*LOC_Os01g12900*), which is thought to promote cell division^39^.

While much is known about the genes that regulate spikelets in rice, no genes or QTLs have been reported for spikelet-related traits in sorghum, except for a few reports of *Multiseeded* genes (*MSD1*-*3*) that control PS fertility^44–46^. To our knowledge, the genetic regulation of spikelet-related organs in sorghum is far behind that of rice.

Recently, we established and reported a sorghum recombinant inbred line (RIL) derived from a parental cross between BTx623 and the Japanese landrace NOG^47^. Each parent seemed to represent diversified sorghum accessions that were classified into three groups; NOG belonged to the subgroup that represented Asian sorghums and was only distantly related to the American/African accessions, including BTx623. We constructed a high-density linkage map based on 3,710 single nucleotide polymorphisms (SNPs) obtained by restriction-site-associated DNA sequencing (RAD-seq) of 213 RIL individuals. This population was suitable for various QTL analyses because, in addition to detecting *Dw3* and *Dw1* as plant height QTLs, other traits showed distinct differences^47,48^. It will be meaningful to improve our knowledge about the sorghum QTLs that control spikelet-related traits and grain size for future sorghum research. In this study, we performed QTL analyses of nine spikelet-related traits and six grain-related traits in sorghum using the RILs to enhance our knowledge of the genetic control of spikelet- and grain-related traits in sorghum.

## Results

### Morphologies of sorghum spikelets, spikelet-related organs, and grain

The panicle of sorghum forms a primary branch at each node, with subsequent secondary or tertiary branches developing from the primary branch^22^. Each inflorescence branch has three terminal spikelets: one sessile spikelet (SS) directly attached to the inflorescence branch and two pedicellate spikelets (PSs) attached to the inflorescence branch via a pedicel (PE). Several spikelet pairs, one SS, and one PS each developed below the terminal spikelets (Fig. 1a,b). Only the SS forms a complete floret consisting of a lemma, palea, two lodicules, three anthers, and a pistil (Fig. 1c–e), and developed into a seed (fertile); whereas, the PS is sterile because it lacks a complete floret structure^49^. Preliminary experiments showed that there were some differences in spikelet-related organs between BTx623 and NOG, and we performed QTL analysis using the RILs derived from a cross between BTx623 and NOG^47^ for genetic dissection of these traits. In addition, to enrich our knowledge of grain size regulation in sorghum, we also performed QTL analysis for grain-related traits. We measured nine spikelet-related traits: sessile spikelet length (SSL), sessile spikelet width (SSW), pedicellate spikelet length (PSL), pedicel length (PEL) (Fig. 1b), anther length (ANL) (Fig. 1d), style length (STYL), stigma length (STIL), stigma width (STIW), and stigma pigmentation (STIP) (Fig. 1e), using spikelets from each RIL collected just before flowering. We also measured six grain-related traits: grain length (GRL), grain width (GRW), grain thickness (GRT) (Fig. 1f), grain length/width ratio (GLWR), grain length/thickness ratio (GLTR), and grain width/thickness ratio (GWTR) of mature seeds in each RIL.

### Phenotypic evaluation of spikelet-related and grain-related traits in the RIL population

**Figure 2:**
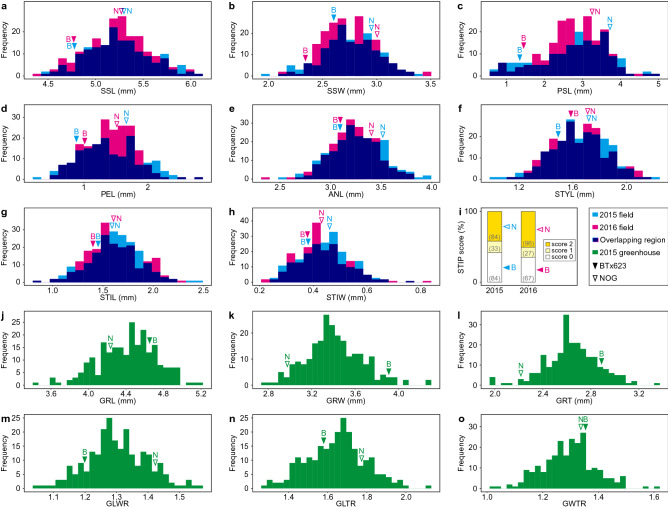
Frequency distribution of nine spikelet-related traits and six grain-related traits in the RIL population. **(a)** Sessile spikelet length (SSL), **(b)** sessile spikelet width (SSW), **(c)** pedicellate spikelet length (PSL), **(d)** pedicel length (PEL), **(e)** anther length (ANL), **(f)** style length (STYL), **(g)** stigma length (STIL), **(h)** stigma width (STIW), **(i)** stigma pigmentation (STIP), **(j)** grain length (GRL), **(k**) grain width (GRW), **(l)** grain thickness (GRT), **(m)** grain length/width ratio (GLWR), **(n)** grain length/thickness ratio (GLTR), and **(o)** grain width/thickness ratio (GWTR). Filled arrowheads (B) and open arrowheads (N) indicate phenotypic values for BTx623 and NOG, respectively.

To validate the reproducibility of our results, the parents (BTx623 and NOG) and RILs in successive generations (F_7_ and F_8_) were cultivated in Tokyo (field) for two consecutive years (2015 and 2016) for the analysis of spikelet-related traits. First, we evaluated the distribution of phenotypic data between experimental cultivations from different years. Focusing on the parents of the RILs, we found that NOG showed higher trait values than BTx623 for all spikelet-related traits (Fig. 2a–i). Although there was variation between years, each phenotype showed a similar distribution, and the phenotypic distribution among the RILs compared to their parents revealed transgressive segregations for most of the traits. For grain-related traits, BTx623 showed higher trait values than NOG with regard to GRL, GRW, and GRT (Fig. 2j–l), indicating that NOG produces smaller grains. No significant difference was detected in GWTR between the parents; however, GLWR and GLTR were larger in NOG, indicating that NOG produces more slender and flatter grains (Fig. 2m–o). Phenotypic distribution among the RILs also showed transgressive segregations for grain-related traits when compared to their parents.

**Figure 3:**
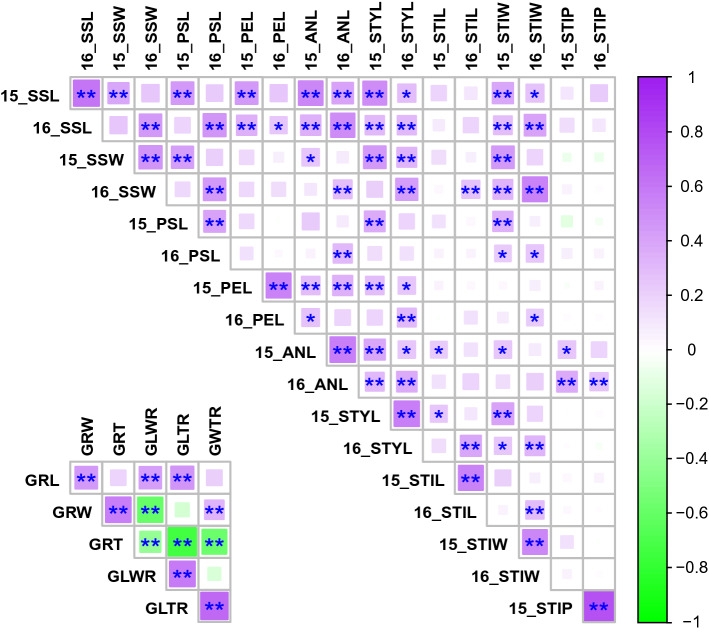
Phenotypic correlations for the nine spikelet-related and six grain-related traits measured in this study. Pearson’s correlation coefficients for each trait measured in the RIL population. Purple and green colors represent positive and negative correlations (*r*) between traits, respectively. *SSL* sessile spikelet length; *SSW* sessile spikelet width; *PSL* pedicellate spikelet length; *PEL* pedicel length; *ANL* anther length; *STYL* style length; *STIL* stigma length; *STIW* stigma width; *STIP* stigma pigmentation; *GRL* grain length; *GRW* grain width; *GRT* grain thickness; *GLWR* grain length/width ratio; *GLTR* grain length/thickness ratio; and *GWTR* grain width/thickness ratio. For the spikelet-related traits, “15_” or “16_” indicate the results from 2015 or 2016. *P < 0.001, **P < 0.0001.

Next, we evaluated the correlation between each trait (Fig. 3). For spikelet-related traits, each dataset was named according to the year and trait name; for example, the SSL phenotypic data collected in 2015 was 15_SSL. Although no negative correlation was observed, significant positive correlations between years were observed for all spikelet-related traits (SSL: *r* > 0.62, *P* < 0.0001; SSW: *r* > 0.49, *P* < 0.0001; PSL: *r* > 0.42, *P* < 0.0001; PEL: *r* > 0.56, *P* < 0.0001; ANL: *r* > 0.59, *P* < 0.0001; STYL: *r* > 0.59, *P* < 0.0001; STIL: *r* > 0.57, *P* < 0.0001; STIW: *r* > 0.55, *P* < 0.0001; and STIP: *r* > 0.78, *P* < 0.0001). We found that STIP had little correlation with other spikelet-related traits. However, STIP was only slightly correlated with ANL. For spikelet-related traits, the correlation among length- and width-related traits tended to exhibit relatively strong correlations except for STIL, suggesting that there are some common genetic mechanisms for the regulation of these spikelet-related traits. For grain-related traits, there were positive correlations between GRL and GRW (r > 0.46, *P* < 0.0001) and GRW and GRT (r > 0.59, *P* < 0.0001).

### QTL analysis of the spikelet-related and grain-related traits in the RIL population

For the genetic dissection of spikelet-related and grain-related traits, we performed QTL analysis using phenotypic data with 3710 SNP markers identified from RAD-seq data^47^. Composite interval mapping was carried out using the data for each trait, and as a result, we identified 36 and 7 QTLs for spikelet-related traits and grain-related traits, respectively (Table 1).

**Table 1:** Summary of the spikelet- and grain-related QTLs detected in this study. Marker intervals were estimated based on confidence intervals (2.0-LOD).

Trait	Year	Chr	QTL ID	Position (cM)	Nearest marker	Marker interval	LOD	PVE (%)	AE
SSL	2015	6	*15_qSSL6*	79.9	Chr06.49894350	Chr06.48086748-Chr06.50331377	4.59	10.17	0.11
		9	*15_qSSL9*	103.8	Chr09.57273964	Chr09.56467290-Chr09.57917278	6.28	14.27	0.13
	2016	6	*16_qSSL6*	78.4	Chr06.48677734	Chr06.48086748-Chr06.50250487	3.92	7.09	0.09
		9	*16_qSSL9*	103.5	Chr09.57166357	Chr09.56467290-Chr09.57917278	8.87	17.03	0.13
SSW	2015	1	*15_qSSW1*	139.9	Chr01.73492838	Chr01.72086062-Chr01.74542240	6.31	9.81	-0.08
		6	*15_qSSW6*	68.3	Chr06.46616258	Chr06.45449430-Chr06.47716228	7.51	11.89	0.09
		7	*15_qSSW7*	114.1	Chr07.64706648	Chr07.63878301-Chr07.65440280	4.63	7.03	-0.07
		9	*15_qSSW9*	99.6	Chr09.56047912	Chr09.54927200-Chr09.56880571	15.09	26.76	0.13
	2016	6	*16_qSSW6*	74.5	Chr06.47921285	Chr06.47546161-Chr06.48677734	6.59	12.13	0.08
		9	*16_qSSW9*	102.0	Chr09.56571248	Chr09.55806970-Chr09.57681027	6.83	12.62	0.09
PSL	2015	6	*15_qPSL6*	70.3	Chr06.46819000	Chr06.45449430-Chr06.47716228	6.50	16.87	0.39
	2016	6	*16_qPSL6*	79.4	Chr06.49336636	Chr06.48086748-Chr06.50331377	5.34	11.73	0.27
PEL	2015	7	*15_PEL7*	86.8	Chr07.59727807	Chr07.59407685-Chr07.60042098	11.38	24.21	0.23
		9	*15_PEL9*	104.2	Chr09.57599415	Chr09.56571248-Chr09.58014412	5.12	9.93	0.15
	2016	7	*16_PEL7*	86.1	Chr07.59407685	Chr07.58788758-Chr07.59727807	9.34	19.53	0.17
ANL	2015	1	*15_qANL1*	165.5	Chr01.78907937	Chr01.77228064-Chr01.79222517	5.10	9.48	0.08
		9	*15_qANL6*	103.5	Chr09.57166357	Chr09.56467290-Chr09.57917278	7.73	14.81	0.10
	2016	1	*16_qANL1*	122.6	Chr01.65134709	Chr01.64608936-Chr01.65571022	4.34	8.35	0.07
		9	*16_qANL9*	103.5	Chr09.57166357	Chr09.56571248-Chr09.57917278	5.92	11.60	0.08
STYL	2015	6	*15_qSTYL6*	76.0	Chr06.48086748	Chr06.47546161-Chr06.48677734	7.35	13.68	0.08
		7	*15_qSTYL7*	86.1	Chr07.59727807	Chr07.59407685-Chr07.60042098	6.69	12.36	0.07
	2016	2	*16_qSTYL2*	116.5	Chr02.67908557	Chr02.67181119-Chr02.68205478	5.35	8.71	-0.06
		6	*16_qSTYL6*	76.0	Chr06.48086748	Chr06.47921285-Chr06.48677734	9.42	16.12	0.08
		7	*16_qSTYL7*	88.1	Chr07.60042098	Chr07.59407685-Chr07.60649532	7.79	13.07	0.07
STIL	2015	1	*15_qSTIL1*	168.8	Chr01.80075044	Chr01.78907937-Chr01.80451024	5.76	9.64	0.08
		3	*15_qSTIL3*	144.2	Chr03.73116039	Chr03.72187849-Chr03.73272999	12.71	23.14	0.12
	2016	1	*16_qSTIL1*	169.0	Chr01.80451024	Chr01.78907937-Chr01.80451024	4.12	6.84	0.07
		3	*16_qSTIL3*	144.2	Chr03.73116039	Chr03.72187849-Chr03.73584465	13.77	25.69	0.14
STIW	2015	3	*15_qSTIW3*	1.2	Chr03.1014391	Chr03.196942-Chr03.1842334	9.59	14.51	-0.04
		6	*15_qSTIW6*	76.0	Chr06.48086748	Chr06.47740711-Chr06.49157376	6.35	9.24	0.03
		9	*15_qSTIW9*	102.0	Chr09.56571248	Chr09.55806970-Chr09.57681027	8.07	11.98	0.03
	2016	3	*16_qSTIW3*	1.2	Chr03.1014391	Chr03.196942-Chr03.1842334	9.07	14.14	-0.04
		6	*16_qSTIW6*	76.3	Chr06.48086748	Chr06.47546161-Chr06.49157376	5.77	8.66	0.03
		9	*16_qSTIW9*	103.5	Chr09.57166357	Chr09.56467290-Chr09.57681027	7.63	11.69	0.03
STIP	2015	1	*15_qSTIP1*	126.6	Chr01.67767009	Chr01.67509497-Chr01.68618079	50.62	68.83	0.77
	2016	1	*16_qSTIP1*	126.6	Chr01.67767009	Chr01.67509497-Chr01.68618079	39.41	61.72	0.72
GRL	2015	4	*qGRL4*	98.6	Chr04.59140841	Chr04.57959040-Chr04.60473635	7.92	16.82	-0.12
GRT	2015	8	*qGRT8*	24.2	Chr08.3019891	Chr08.3019891-Chr08.4069903	5.43	11.86	-0.08
GLWR	2015	8	*qGLWR8*	50.2	Chr08.6382451	Chr08.5764378-Chr08.7916525	3.77	8.39	0.03
GLTR	2015	4	*qGLTR4*	96.1	Chr04.57959040	Chr04.57115462-Chr04.58794505	15.92	28.84	-0.08
		8	*qGLTR8.1*	23.6	Chr08.3019891	Chr08.2790929-Chr08.3185552	3.49	5.71	0.03
		8	*qGLTR8.2*	83.3	Chr08.57258936	Chr08.56896649-Chr08.57745670	3.99	6.52	0.03
GWTR	2015	4	*qGWTR4*	96.1	Chr04.57959040	Chr04.57115462-Chr04.58794505	6.44	13.92	-0.04

*PVE* phenotypic variance explained. For the additive effects (AE), positive values indicate that alleles from NOG increased the trait score. *SSL* sessile spikelet length, *SSW* sessile spikelet width, *PSL* pedicellate spikelet length, *PEL* pedicel length, *ANL* anther length, *STYL* style length, *STIL* stigma length, *STIW* stigma width, *STIP* stigma pigmentation, *GRL* grain length, *GRT* grain thickness, *GLWR* grain length/width ratio, *GLTR* grain length/thickness ratio, *GWTR* grain width/thickness ratio.

### Spikelet-related traits

**Figure 4:**
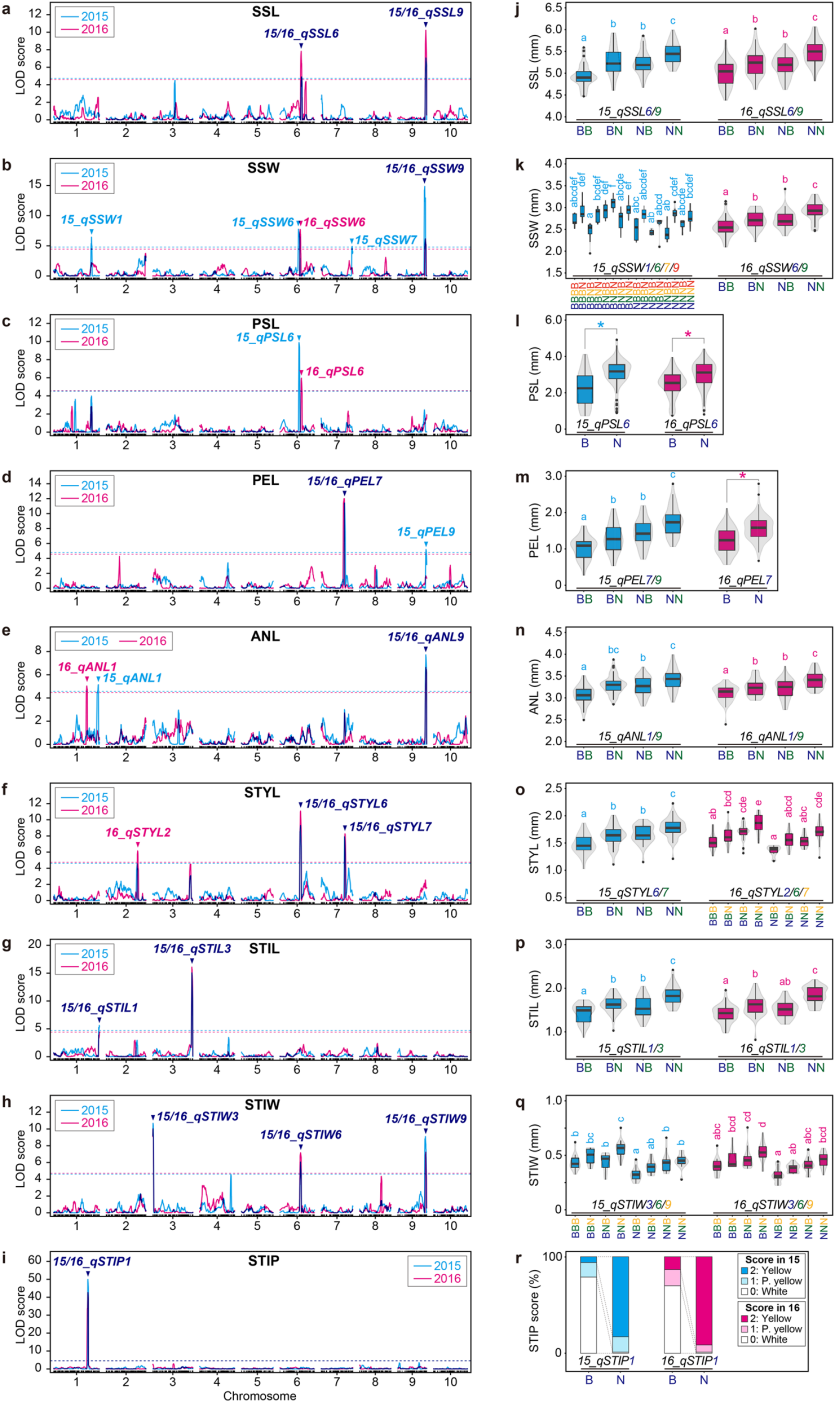
Results of QTL analysis for the nine spikelet-related traits **(a–i)** and their allelic effects **(j–r)**. **(a,j)** Sessile spikelet length, **(b,k)** sessile spikelet width, **(c,l)** pedicellate spikelet length, **(d,m)** pedicel length, **(e,n)** anther length, **(f,o)** style length, **(g,p)** stigma length, **(h,q)** stigma width, and **(i,r)** stigma pigmentation. **(a–i)** LOD profiles obtained from composite interval mapping (CIM). Cyan and magenta horizontal dotted lines represent a threshold of the 1000 × permutation test (*P* < 0.05) in 2015 and 2016, respectively. **(j–r)** Contributions of SNP genotypes for significant QTLs. Box and violin plots **(j–q)** and a 100% stacked column chart (r) show the effects of the nearest marker genotypes for each QTL or allelic combinations of QTLs. “15_” or “16_” indicate the results from 2015 or 2016. Different letters denote significant differences according to the Tukey–Kramer test (*P* < 0.05). Asterisks indicate significant differences between genotypes (Welch’s *t*-test, *P* < 0.01).

For sessile spikelet length (SSL), two QTLs were detected on chromosomes 6 and 9 in both 2015 and 2016 trials (15_*qSSL6*, 15_*qSSL9*, 16_*qSSL6*, and 16_*qSSL9*) with the logarithm of the odds (LOD) scores ranging from 3.92 to 8.87, and the percentage of phenotypic variation explained by each QTL (PVE) values from 7.09 to 17.03% (Fig. 4a, Table 1). Comparisons of confidence intervals (2.0-LOD) of each QTL between 2015 and 2016 suggested that *qSSL6* and *qSSL9* were highly reproducible and reliable QTLs. *qSSL9* had a higher LOD score (6.28 in 2015, 8.87 in 2016) and was relatively robust in each cultivation compared to *qSSL6*. The effects of increasing SSL were attributed to the NOG alleles for *qSSL6* and *qSSL9* (Fig. S1a, Fig. 4j).

For sessile spikelet width (SSW), four QTLs were detected on chromosomes 1, 6, 7, and 9 in 2015 (15_*qSSW1*, 15_*qSSW6*, 15_*qSSW7*, and 15_*qSSW9*), and two QTLs were detected on chromosomes 6 and 9 in 2016 (16_*qSSW6* and 16_*qSSW9*), with LOD scores ranging from 4.63 to 15.09, and PVE values from 7.03% to 26.76% (Fig. 4b, Table 1). Comparisons of confidence intervals suggested that *qSSW9* had high reproducibility, while the other QTLs were more susceptible and unstable due to environmental factors. *qSSW9* had a higher LOD score (15.09 in 2015 and 6.83 in 2016) and was relatively robust in each cultivation compared with the other QTLs. The effects of increasing SSW were attributed to the NOG alleles for 15/16_*qSSW6* and 15/16_*qSSW9*, but to the BTx623 allele for 15_*qSSW1* and 15_*qSSW7* (Fig. S1b, Fig. 4k).

For pedicellate spikelet length (PSL), a single QTL was detected on chromosome 6 in 2015 (15_*qPSL6*; LOD score 6.50, PVE 16.87%) and 2016 (16_*qPSL6*; LOD score 5.34, PVE 11.73%) (Fig. 4c, Table 1). Although two QTLs were detected in close proximity, their confidence intervals did not overlap, suggesting that PSL is unstable in response to environmental factors. The effects of increasing PSL were attributed to the NOG alleles for both 15_*qPSL6* and 16_*qPSL6* (Fig. 4l).

For pedicel length (PEL), two QTLs were detected on chromosomes 7 and 9 in 2015 (15_*qPEL7* and 15_*qPEL9*), and a single QTL was detected on chromosome 7 in 2016 (16_*qPEL7*) with LOD scores ranging from 5.12 to 11.38 and PVE values from 9.93 to 24.21% (Fig. 4d, Table 1). Although the peak markers were slightly different, 15_*qPEL7* and 16_*qPEL7* had overlapping confidence intervals, suggesting that *qPEL7* is a reproducible and reliable QTL. The effects of increasing PEL were attributed to the NOG alleles at all QTLs (Fig. S1c, Fig. 4m).

For anther length (ANL), two QTLs were detected on chromosomes 1 and 9 in both 2015 and 2016 (15_*qANL1*, 15_*qANL9*, 16_*qANL1*, and 16_*qANL9*), with LOD scores ranging from 4.34 to 7.73 and PVE values from 8.35% to 14.81% (Fig. 4e, Table 1). Comparisons of confidence intervals suggested that *qANL9* showed high reproducibility, while *qANL1* was unstable to environmental factors. The effects of increasing ANL were attributed to the NOG alleles for all QTLs (Fig. S1d, Fig. 4n).

For style length (STYL), two QTLs were detected on chromosomes 6 and 7 in 2015 (15_*qSTYL6* and 15_*qSTYL7*), and three QTLs were detected on chromosomes 2, 6, and 7 in 2016 (16_*qSTYL2*, 16_*qSTYL6*, and 16_*qSTYL7*) with LOD scores ranging from 5.35 to 9.42, and PVE values from 8.71% to 16.12% (Fig. 4f, Table 1). Both 15/16_*qSTYL6* and 15/16_*qSTYL7* had overlapping confidence intervals, suggesting that *qSTYL6* and *qSTYL7* are robust and reliable QTLs, whereas *qSTYL2* is unstable to environmental factors. The effects of increasing STYL were attributed to the NOG alleles in 15/16_*qSTYL6* and 15/16_*qSTYL7*, but to the BTx623 allele in 16_*qSTYL2* (Fig. S1e, Fig. 4o).

For stigma length (STIL), two QTLs were detected on chromosomes 1 and 3 in both 2015 and 2016 (15_*qSTIL1*, 15_*qSTIL3*, 16_*qSTIL1*, and 16_*qSTIL3*) with LOD scores ranging from 4.12 to 13.77 and PVE values from 6.84% to 25.69% (Fig. 4g, Table 1). Both 15/16_*qSTIL1* and 15/16_*qSTIL3* had overlapping confidence intervals, suggesting that *qSTIL1* and *qSTIL3* are highly reproducible and reliable QTLs. The effects of increasing STIL were attributed to the NOG alleles for all QTLs (Fig. S1f, Fig. 4p).

For stigma width (STIW), three QTLs were detected on chromosomes 3, 6, and 9 in both 2015 and 2016 (15_*qSTIW3*, 15_*qSTIW6*, 15_*qSTIW9*, 16_*qSTIW3*, 16_*qSTIW6*, and 16_*qSTIW9*) with LOD scores ranging from 5.77 to 9.59, and PVE values from 8.66% to 14.51% (Fig. 4h, Table 1). Comparisons of the confidence intervals suggested that *qSTIW3*, *qSTIW6*, and *qSTIW9* are highly reproducible and reliable QTLs. The effects of increasing STIW were attributed to the NOG alleles for *qSTIW6* and *qSTIW9* and to the BTx623 allele for *qSTIW3* (Fig. S1g, Fig. 4q).

For stigma pigmentation (STIP), an extremely significant QTL was detected on chromosome 1 in both 2015 (15_*qSTIP1*; LOD score 50.62, PVE 68.83%) and 2016 (16_*qSTIP1*; LOD score 39.41, PVE 61.72%) (Fig. 4i, Table 1). The complete match of the confidence intervals for 15_*qSTIP1* and 16_*qSTIP1* indicated that *qSTIP1* is a highly reproducible and reliable QTL. The effect of increasing STIP (to be yellow) was attributed to the NOG alleles for *qSTIP1* (Fig. 4r)*.*

### Grain-related traits

**Figure 5:**
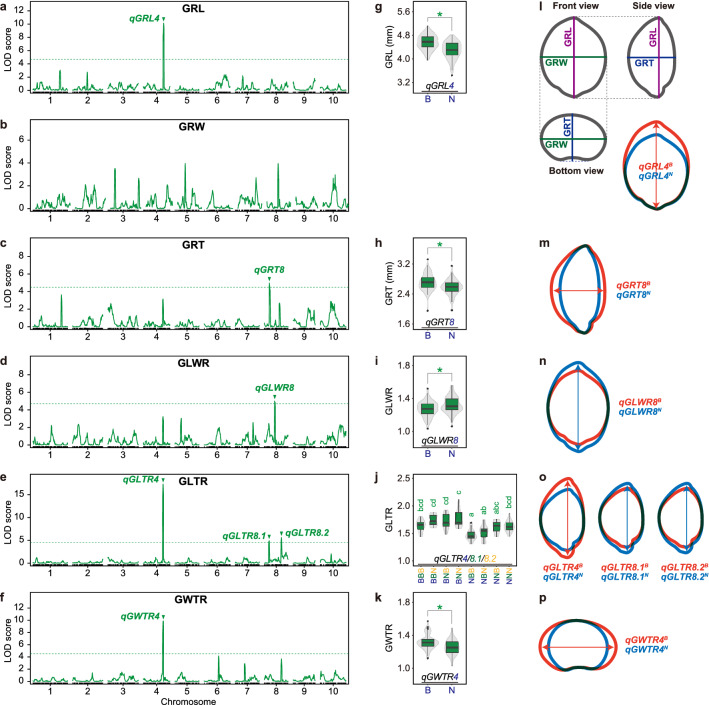
Results of QTL analysis for the six grain-related traits **(a–f)**, their allelic effects **(g–k)**, and schematics of the allelic effects **(l-p)**. **(a,g,l)** Grain length, **(b)** grain width, **(c,h,m)** grain thickness, **(d,i,n)** grain length/width ratio, **(e,j,o)** grain length/thickness ratio, and **(f,k,p)** grain width/thickness ratio. **(a–f)** LOD profiles obtained from composite interval mapping (CIM). Horizontal dotted lines represent a threshold of the 1000 × permutation test (*P* < 0.05). **(g–k)** Contributions of SNP genotypes for significant QTLs. Box and violin plots show the effects of the nearest marker genotypes for each QTL or allelic combinations of QTLs. Different letters denote significant differences according to the Tukey–Kramer test (*P* < 0.05). Asterisks indicate significant differences between genotypes (Welch’s *t*-test, *P* < 0.01).

A single QTL on chromosome 4 (*qGRL4*; LOD score 7.92, PVE 16.82%) was detected for grain length (GRL) (Fig. 5a, Table 1). The effect of increasing GRL was attributed to the BTx623 allele (Fig. 5g,l). Although we could not identify any QTLs for grain width (Fig. 5b), a single QTL was detected for grain thickness (GRT) on chromosome 4 (*qGRT8*; LOD score 5.43, PVE 11.86%) (Fig. 5c, Table 1). The effect of increasing GRT and GRL effects was attributed to the BTx623 alleles (Fig. 5h,m).

For the grain length/width ratio (GLWR), a single QTL was detected on chromosome 8 (*qGLWR8*; LOD score 3.77, PVE 8.39%) (Fig. 5d, Table 1). The effect of increasing GLWR was attributed to the NOG allele (Fig. 5i,n). For the grain length/thickness ratio (GLTR), three QTLs were detected on chromosomes 4 and 8 (*qGLTR4*, *qGLTR8.1*, *and qGLTR8.2*) with LOD scores ranging from 3.49 to 15.92 and PVE values from 5.71% to 28.84% (Fig. 5e, Table 1). The effects of increasing GLTR were attributed to the BTx623 alleles for *qGLTR4* and to the NOG allele for *qGLTR8.1* and *qGLTR8.2* (Fig. S1h, Fig. 5j,o).

For the grain width/thickness ratio (GWTR), a single QTL was detected on chromosome 4 (*qGWTR4*; LOD score 6.44, PVE 13.92%) (Fig. 5f, Table 1), and the effect of increasing GWTR was attributed to the BTx623 allele (Fig. 5k,p).

**Figure 6:**
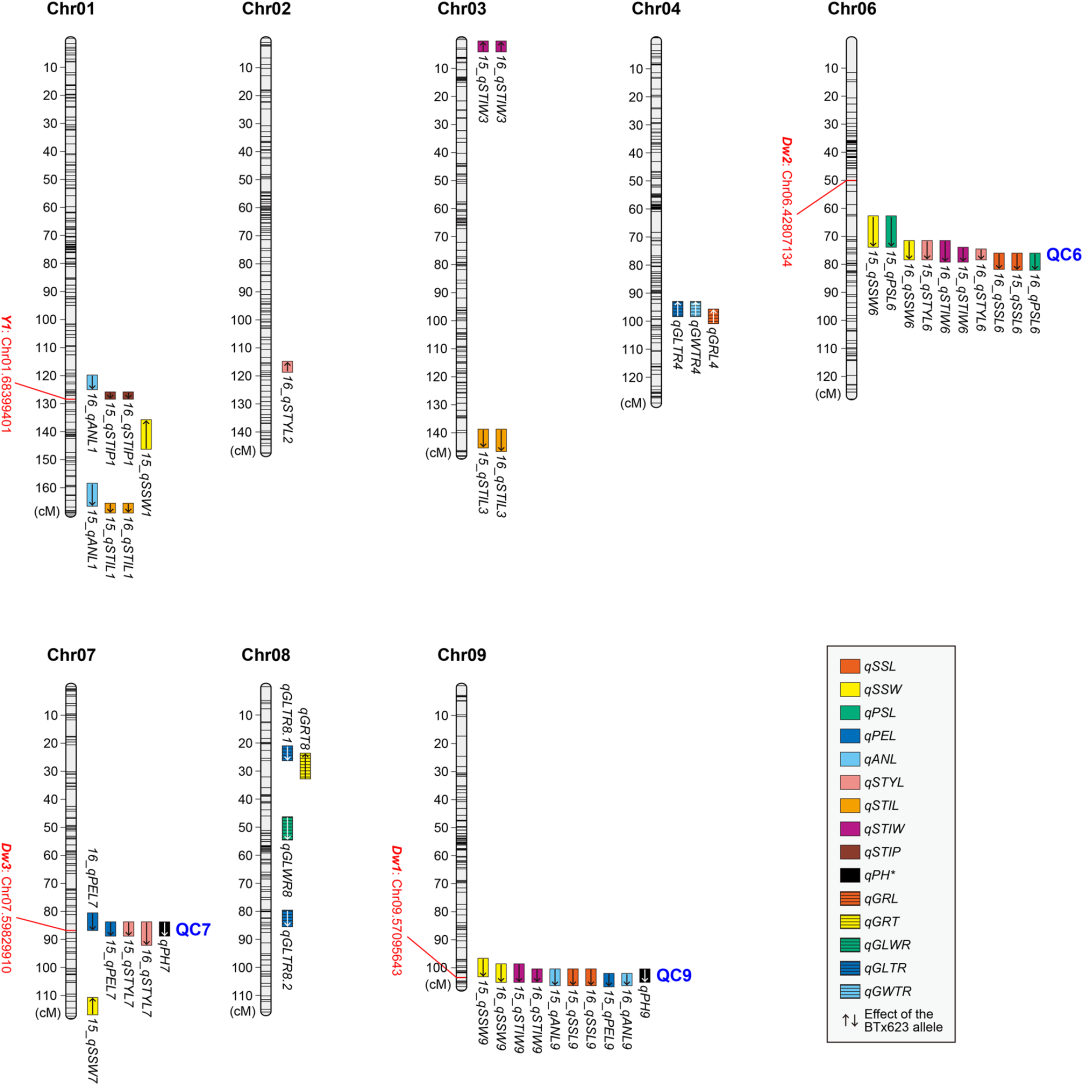
Graphical representations of the QTLs detected in this study. Colored boxes indicate the confidence intervals (2.0-LOD) of significant QTLs, with each color representing a different trait. Arrows in each box indicate the direction of the phenotypic effect of the BTx623 allele (up, increasing; down, decreasing). *Positions of *qPH7/9* were taken from the results obtained by our previous study using the same population^47^. For the spikelet-related traits, “15_” or “16_” indicate results from 2015 or 2016. QC6, QC7, and QC9 indicate QTL clusters found on chromosomes 6, 7, and 9, respectively. Transcriptional start points for known related genes in BTx623 are shown in red on the left side of the chromosome.

The positions of the confidence intervals on the chromosomes for all the QTLs detected in this study are summarized in Fig. 6. The detected QTLs were distributed across eight chromosomes, which did not include chromosomes 5 and 10. QTL clusters with four or more QTLs detected in similar positions were found on chromosomes 6, 7, and 9 (QC6, QC7, and QC9 in Fig. 6). Among these QTL clusters, QC6 affected SSL, SSW, PSL, STYL, and STIW; QC7 affected PEL and STYL; and QC9 affected SSL, SSW, PEL, ANL, and STIW.

### Comparison between our QTLs and those of previous studies and a search for the responsible genes

In our QTL analysis of nine spikelet-related traits, three QTL clusters were identified on chromosomes 6 (QC6, Chr06:47546161–50331377), 7 (QC7, Chr07:58788758–60649532), and 9 (QC9, Chr09:54927200–57917278). Whether the formation of these QTL clusters was due to the pleiotropic effects of a single gene or simply the physical linkage of multiple genes is unclear; however, it will be interesting to identify the genes responsible for these QTL clusters.

For QC7 and QC9, each cluster corresponded to plant height-related QTLs obtained in our previous report using the same populations and genotype data^47^ (Fig. 6; *qPH7* and *qPH9*). Comparing these QTLs with those reported previously using the QTL Atlas^50^, both QC7 and QC9 were supported by numerous previous reports of plant height- and panicle length-related QTLs in sorghum^27,51–57^ (Table S1). In sorghum, four major loci controlling plant height, *Dw1*, *Dw2*, *Dw3*, and *Dw4*, have been extensively characterized and reported that their recessive alleles reduce internode length^6^. The *Dw1* locus was mapped to chromosome 9, *Dw2* to 6, *Dw3* to 7, and *Dw4* to 4^58^. *Dw1* (Sobic.009G230800, Chr09:57093313–57095643), which is known to regulate the length of internodes by controlling cell proliferation, encodes a positive regulator gene of brassinosteroid signaling^7,8,59^. *Dw2* (Sobic.006G067700, Chr06:42803037–42807134) encodes a protein kinase^9^, and *Dw3* (Sobic.007G163800, Chr07:59821905–59829910) encodes an ABCB1 auxin efflux transporter^10^. In addition to internode length, pleiotropic effects of *Dw3*, including seed weight, panicle size, tiller number, and leaf angle, have also been reported^60–62^. We previously showed that *Dw1* and *Dw3* are likely the genes responsible for *qPH9* and *qPH7* based on sequencing analysis, which revealed that NOG had functional alleles of both *Dw1* and *Dw3* genes, while BTx623 had loss-of-function alleles of both genes^47^ (Fig. S2a and b). Based on the functional aspects of *Dw1* and *Dw3* (e.g., controlling organ size) and the direction of the allelic effects, in which the size of spikelet-related organs and plant height were increased in the NOG allele, QC7 and QC9 likely corresponded to *Dw3* and *Dw1,* respectively.

For the gene responsible for QC6, we initially hypothesized that *Dw2* is also involved in this cluster because *Dw2* is located on chromosome 6^9^. However, *Dw2* was found to be located much further upstream than the QC6 region (*Dw2*, Chr06:42803037–42807134; QC6, Chr06:47546161–50331377; Fig. 6). In addition, the lack of QTLs detected around the *Dw2* region in our previous QTL analysis for plant height using the same populations^47^ indicated that there were no functional differences in *Dw2* between the parents of our RILs (BTx623 and NOG). Therefore, we concluded that *Dw2* was not responsible for QC6. Although no organ size-related genes have been identified around the QC6 region to date, we searched for reported QTLs around QC6 using the QTL Atlas (Table S1). As a result, QTLs for plant height^53,63^, fresh biomass^64^, and panicle length^65,66^ were found within the QC6 region, suggesting that minor organ size-related gene(s) other than the major *Dw* genes are located in the QC6 region.

For *qSTIP1*, we found that QTLs for grain color and proanthocyanidins were reported around this locus^67,68^ (Table S1). Furthermore, we also found that the *Y1* gene (Sobic.001G398100, Chr01:68399401–68400602), an MYB transcription factor that controls seed pericarp color by regulating flavonoid biosynthesis^69,70^, is encoded within the confidence interval of *qSTP1*. *Y1* is the ortholog of maze *P1* (Pericarp color 1), a transcriptional regulator involved in flavonoid-related red pigmentation in the maize pericarp^71^. Given *Y1* as a strong candidate for *qSTIP1*, we confirmed polymorphisms of the *Y1* locus between BTx623 and NOG by PCR and sequencing in this study. Consistent with the previously reported pattern of functional and loss-of-function alleles^70^, BTx623 has a loss-of-function allele with a 3.2 kb deletion, while NOG has a functional allele with no deletion (Fig. S2c). These results suggest that *Y1* is responsible for both seed and stigma colors.

For spikelet-related traits, all other QTLs detected in this study were explained in previous reports. For the confidence interval of 16_*qANL1*, the QTLs for panicle length^22^ and dry matter growth^72^; 15_*qSSW1*, the QTLs for fresh biomass^73^, grain weight^74^, and panicle width^75^; 15_*qANL1*, the QTLs for leaf length and panicle length^26^; 15_*qSTIL1*, the QTLs for panicle width^76^ and fresh biomass^51^; 16_*qSTYL2*, the QTLs for plant height^53^ and panicle length^17^; 15_*qSTIW3*, the QTLs for shoot length^77^ and panicle length^78^; 15_*qSTIL3*, the QTL for stem circumference^79^; 15_*qPSL6*, the QTLs for plant height^63,80^ and panicle length^25^; and 15_*qSSW7*, the QTLs for panicle length and grain yield^25^ have been reported (Table S1). Hence, QTLs for organ size regulators have been reported within the confidence interval of the length- and width- related QTLs detected in this study, confirming the reliability of our results. However, the details of these QTLs remain unclear, and further research is required to identify the genes and corresponding mutations responsible for these traits.

We tested whether sorghum orthologous genes of the spikelet-related organ regulator genes identified in rice could be responsible for the QTLs detected in our analysis. Putative orthologous genes in sorghum were defined using BLASTP results and synteny information. The putative orthologue of *GS3*: Sobic.001G341700 (Chr01:62910779–62916258), *GW5*: Sobic.009G070000 (Chr09:8184500–8186318), *SG6*: Sobic.010G220800 (Chr10:56310472–56315161), *qSTL3* (*LOC_Os03g14850*): Sobic.007G086200 (Chr07:11383927–11384670), *OsSYL2*: Sobic.004G247700 (Chr04:59522591–59523507), and *OsRac1*: Sobic.009G084100 (Chr09:13296136–13300901) were identified from our analysis. However, we found that none of the putative orthologs of the spikelet-related organ regulator genes in rice were included in the confidence intervals of the QTLs for sorghum spikelet-related traits (Table 1 and Table S1).

We expected that *Multiseeded* genes (*MSD1*-*3*)^44–46^ may affect PSL, however, no QTLs were detected around *MSD1* (Sobic.007G135700, Chr07: 56156125–56157410), a TCP transcription factor^44^; and *MSD3* (Sobic.001G407600, Chr01:69162600..69165731), a plastidial ω-3 fatty acid desaturase^46^. On the other hand, we found that *MSD2* (Sobic.006G095600, Chr06: 46567412–46571064), a lipoxygenase^45^, was encoded within the confidence interval of 15_*qPSL6* (Table 1 and Table S1). However, there were no polymorphisms in the ORF of *MSD2* between the parents of our RILs (data not shown).

For the grain-related traits, all the QTLs detected in this study were also supported by previous studies that reported grain size and weight QTLs^17–21^ (Table S1). We found that orthologous genes for rice *PGL2* (Sobic.004G237000, Chr04: 58488864–58490438) and maize *Gln-4* (Sobic.004G247000, Chr04: 59472640–59476805) were encoded within the confidence interval of *qGRL4*. *PGL2* encodes an atypical bHLH protein that controls grain length and weight in rice^81^, while *Gln-4* encodes a cytosolic glutamine synthetase related to seed weight in maize^82^. We expected that *PGL2* or *Gln4* orthologs in sorghum would be responsible for *qGRL4*; however, there were no polymorphisms in ORFs in either gene between the parents of our RILs (data not shown). The *OsSYL2* ortholog (Sobic.004G247700) is also encoded within the confidence interval of *qGRL4*; however, there were no polymorphisms in the ORF of the gene between the parents of our RILs (data not shown).

## Discussion

Phenotypic distributions among the RILs showed transgressive segregation for almost all of the traits, suggesting a polygenic inheritance of spikelet- and grain-related traits in sorghum. As expected, multiple QTLs were detected for many of these traits; however, considering the phenotypic distribution pattern and sum of PVE values, it is likely that there are still quite a few minor QTLs that could not be detected in this study.

We initially expected that orthologs of genes known to regulate spikelet-related organs in rice would also be involved in the regulation of the sorghum spikelet; however, no such orthologs were detected as QTLs in this population. These results suggest that rice and sorghum may utilize widely different systems to control spikelet-related traits. However, further research using a large number of sorghum varieties is required to validate this hypothesis, since the present results were obtained from analyses using only a single biparental population. For PSL and the grain-related traits, we found that *MSD2* was encoded within the confidence interval of 15_*qPSL6*; and orthologous genes for rice *PGL2* and maize *Gln-4* were encoded within the confidence interval of *qGRL4*. Although there were no polymorphisms in ORFs in either gene between the parents of our RILs, further investigation is needed to determine whether these orthologs are involved in sorghum pedicellate spikelet length and grain size, as polymorphisms in the promoter regions of these genes may also alter these traits.

Because we could not measure the spikelet- and grain-related traits under the same environment in this study, we cannot conclusively discuss the correlation between these traits; however, we examined it only as a guide. In our population, we found that grain- and spikelet-related traits showed weak correlations, except for SSW and STIW (Fig. S3). Based on these results and the fact that the QTLs detected for spikelet-related traits and grain-related traits were completely different (Figs. 4, 5, 6), we hypothesized that spikelet size and grain size are not mutually associated in our sorghum population, unlike what has been reported in rice^42^, barley^83,84^, and wheat^85,86^. These differences might be due to both parents of our population belonging to the “grain sorghum type” in which the grains protrude from the glumes; thus, grain size may not be limited by spikelet (glume) size.

It is noteworthy that *Dw3* and *Dw1*, which have significant effects on the spikelet-related organ size, were not detected for grain-related traits in our population, even though *dw3* was previously reported to reduce grain yield by reducing grain size in sorghum^87^. Similarly, RNAi-mediated repression of the *Dw1* ortholog reduces grain size in rice^59^, but it has not yet been reported whether *Dw1* affects grain size in sorghum. To validate these points, we re-examined whether *Dw3* and *Dw1* affect the grain size parameters (GRL, GRW, and GRT) (Fig. S4). We found that both *Dw3* and *Dw1* had little or no effect on the grain size parameters. For the *Dw1* allele, grain length and thickness were larger in the functional allele (*Dw1*); however, the grain width was larger in the *dw3* mutant allele than in the functional *Dw3* allele (Fig. S4a). Contrary to previous reports^87^, *dw3* had no effect on the grain length and thickness but rather increased the grain width, suggesting that *dw3* does not reduce grain size in this population. We showed that grain length and thickness slightly decreased in *dw1*, which is consistent with the results reported in rice^59^. This result suggests that a reduction in grain size in *dw1* may reduce grain yield. Considering that *dw3* and *dw1* have been widely used in grain sorghum breeding, we hypothesized that the degree of reduction in grain size (disadvantage) due to *dw3* and *dw1* was much smaller than the degree of reduction in plant height (advantage), which is why *dw3* and *dw1* are convenient for breeding programs. We also checked the influence of *dw3* and *dw1* on plant height and found that they reduced plant height by 45.8% and 32.8%, respectively, but the reduction in grain size was at most 3% (Fig. S4b). These results support our hypothesis in this study.

By enriching the knowledge about the genetic regulation of spikelet- and grain-related traits using a large number of sorghum varieties in the future, we may be able to improve these traits without worrying about trade-offs; for example, breeders may be able to improve grain-related traits of elite varieties of grain sorghum without changing their plant height by introducing grain-related QTLs, such as *qGRL4* and *qGRT8*, which are not related to plant height.

The QTLs identified in this study will be informative for understanding the genetic basis of spikelet-related organ morphologies and future breeding of spikelet- and grain-related traits in sorghum.

## Methods

### Plant materials

Recently, we established and reported a sorghum recombinant inbred line (RIL), derived from a parental cross between BTx623 and the Japanese landrace NOG^47^. This population was suitable for various QTL analyses because, in addition to detecting *Dw3* and *Dw1* as plant height QTLs, other traits showed distinct differences^47,48^. Therefore, we believe that this population might also be suitable for the analysis of spikelet- and grain-related traits in this study. Seeds of BTx623 were kindly provided by John Mullet and Bill Rooney of Texas A&M University. NOG seeds, originally from Iwate Prefecture, Japan, were purchased from Noguchi Seeds (Hannou, Saitama, Japan). In total, 213 RILs were generated by recurrent selfing of progeny derived from a cross with BTx623 and NOG at the Institute of Plant Science and Resources (IPSR) at Okayama University, as previously reported^47^. All seed stocks were stored at 15 °C until further use. All applicable international, national, and institutional guidelines for the use of plants in the present study were followed.

### Genotype data

Genotype data of the RILs were obtained using the restriction site-associated DNA sequencing (RAD-seq) method^88^. Briefly, each genome of the 213 RILs from the F_6_ generation was subjected to RAD-seq to detect genome-wide SNPs, and 3710 high-quality SNPs were selected to construct the linkage map. The map covered a total length of 1299.7 cM and had an average marker density of 0.4 cM. Detailed methods and information for genotyping have been described in our previous reports^47,89^.

### Cultivation

Field trials were performed in a green field at the University of Tokyo (latitude: 35°42′58.5′′ N, longitude: 139°45′44.5′′ E), Yayoi, Bunkyo-Ku, Tokyo, Japan, from May to September in 2015 and 2016. Plants were germinated in 200-cell trays filled with synthetic culture soil (BONSOL 1, Sumitomo Chemical Co., Ltd., Japan) for two weeks, and then transplanted into the field (n = 2 per line). The space between ridges was 60 cm, with a 15 cm distance between individuals in the same ridge. The field was fertilized using N:P:K = 10:10:10 (kg ha^–1^) fertilizer before the trials in each year. Cultivation and phenotyping for spikelet-related traits were conducted in 2015 and 2016, using the F_7_ and F_8_ generations, respectively. For grain-related traits, cultivation was performed in an open-air greenhouse at the experimental farm of the IPSR (latitude: 34°35′30.5′′ N, longitude: 133°46′08.2′′ E) in Kurashiki, Okayama, Japan, from June to September 2015 with the F_7_ generation. Plants were germinated in small trays filled with vermiculite for approximately two weeks and then transplanted to 30-cm diameter pots filled with soil from the IPSR field (n = 2 per line). Mature grains (F_8_ seeds) were harvested from one healthy plant per line (F_7_ generation) to phenotype grain-related traits.

### Phenotyping

Sessile spikelet length (SSL), sessile spikelet width (SSW), pedicellate spikelet length (PSL), pedicel length (PEL), anther length (ANL), style length (STYL), stigma length (STIL), stigma width (STIW), and stigma pigmentation (STIP) were measured. To standardize the developmental stages of spikelets among the RILs, we sampled spikelets at the just-before-flowering stage in each RIL. In the inflorescence that began flowering at the top, we defined spikelets at the just-before-flowering stage (spikelets that would flower within a day) as those attached to the primary branch just below the flowered primary branch. In each RIL, we sampled three second-top spikelet pairs (Fig. 1a arrow, one SS, and one PS each) on the secondary branches developing in the middle portion of the inflorescence from one healthier plant per line at the just-before-flowering stage. Sampled spikelets were dissected and photographed under a stereomicroscope (M125, Leica, Germany) with a CCD camera (MC170 HD, Leica). Length—and width-related traits were measured using Photoshop CS5 (Adobe, USA). The definitions of each length- and width-related trait were as follows: SSL, length of SS (outer glume) from base to tip, SSW: length of the part of the SS where the width of the SS was the largest, PSL: length of PS from base to tip, PEL: length of PE from base to the beginning of PS, ANL: longitudinal length of AN, STYL: length of STY from the upper end of the ovary to the beginning of STI; STIL, longitudinal length of STI; and STIW, length of the STI, where the width of the STI was the largest. We subjectively scored STIP values on a three-step scale, with white as 0, intermediate color (pale yellow) as 1, and yellow as 2. For all spikelet-related traits, the average value of three independent spikelets was used as the phenotypic value of an individual plant.

Grain length (GRL), grain width (GRW), and grain thickness (GRT) were measured. We used mature grains collected from the whole inflorescence of a single individual for analysis. Briefly, we captured an image of 10 mature grains for each RIL using a flatbed scanner (GT-X820, EPSON, Japan), and quantified these three traits as grain size parameters using the MATLAB Image Processing Toolbox (MATLAB_R2015a). Detailed methods and information for the acquisition and analysis of grain images have been described in our previous reports^90^. The grain length/width ratio (GLWR), grain length/thickness ratio (GLTR), and grain width/thickness ratio (GWTR) were calculated from the quantified GRL, GRW, and GRT. Correlation analysis was performed using the R^91^/corrplot package^92^ and modified using Illustrator CS5 (Adobe).

### QTL mapping

Genotype probabilities were calculated using the calc.genoprobility function with a step size of 1 cM and an assumed genotyping error probability of 0.05, using the Kosambi map function^93^ as implemented in the R/qtl package^94^. QTL analysis was performed using the composite interval mapping (CIM) function of the R/qtl package with the Haley–Knott regression method^95^. Linkage analysis was performed using the R/qtl package in R version 3.5.2. The LOD significance threshold for detecting QTLs was calculated by performing 1000 iterations using the R/qtl permutation test. Confidence intervals (CIs) for the QTLs were estimated based on the 2.0-LOD support interval, and the nearest flanking markers located outside the boundary of each CI were defined as both ends of the marker intervals. The additive effect and the percentage of phenotypic variance explained (PVE) by each QTL were obtained using the fitqtl function of the R/qtl package. Box and violin plots were created using the R/ggplot2 package^96^. A graphical representation of QTLs with linkage groups and markers was produced using the MapChart software package^97^ and modified using Illustrator CS5.

### Comparison between our QTLs and those of previous studies and a search for the responsible genes

Comparisons between detected QTLs in this study and previously reported sorghum QTLs were performed using the QTL Atlas^50^ (https://aussorgm.org.au/sorghum-qtl-atlas/). To search for sorghum orthologs of rice or maize responsible genes, we used the BLASTP program in the Phytozome database^98^ (https://phytozome.jgi.doe.gov/pz/portal.html) and synteny information obtained from SynMap2^99^ (http://genomevolution.org/coge/SynMap.pl). We defined the sorghum genes supported by both BLASTP results (< E^-10^) and syntenic information as putative orthologs of these genes. PCR and subsequent sequencing were performed according to previous reports^8,70^. Primers used for the validation of polymorphisms in the candidate genes are listed in Supplementary Table S2.

## Supplementary Information

Supplementary Information.
