# Supplementary material for: Genetic dissection of QTLs associated with spikelet-related traits and grain size in sorghum
Source: Sci Rep. 2021 Apr 30;11:9398. doi: 10.1038/s41598-021-88917-x (PMC8087780; doi:10.1038/s41598-021-88917-x)
Supplement: Supplementary file 1 — Supplementary Information. [file 41598_2021_88917_MOESM1_ESM.pdf]

Supplementary Information

Title:

Genetic dissection of QTLs associated with spikelet-related traits and grain size in sorghum

Authors:

Hideki Takanashi[1,*], Mitsutoshi Shichijo[1], Lisa Sakamoto[1], Hiromi Kajiya-Kanegae[1,#], Hiroyoshi Iwata[1], Wataru Sakamoto[2], Nobuhiro Tsutsumi[1,*]

[1]Graduate School of Agricultural and Life Sciences, The University of Tokyo, 1-1-1 Yayoi, Bunkyo-ku, Tokyo 113-8657, Japan

[2]Institute of Plant Science and Resources, Okayama University, Kurashiki, Okayama 710-0046, Japan

[#]Present address: Research Center for Agricultural Information Technology, National Agriculture and Food Research Organization, Chiyoda-ku, Tokyo 100-0013, Japan

*Co-corresponding authors

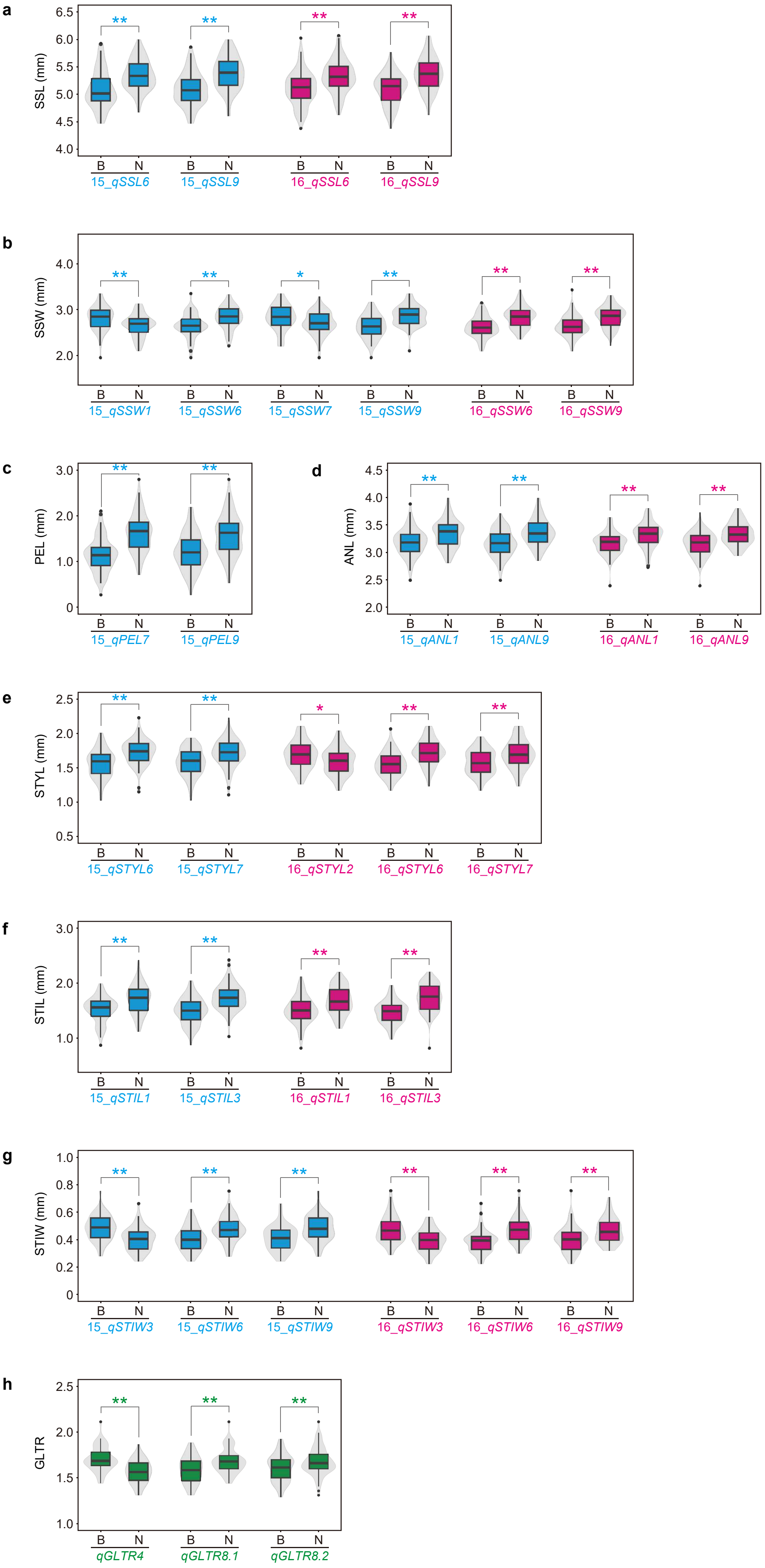

Supplementary Figure S1

Box and violin plots show the effects of the nearest marker genotypes for each QTL. (a) Sessile spikelet length, (b) sessile spikelet width, (c) pedicel length, (d) anther length, (e) style length, (f) stigma length, and (g) stigma width. “15_” or “16_” indicate cultivation in 2015 or 2016. Asterisks indicate significant differences between genotypes (Welch’ s t-test, *P < 0.05, **P < 0.005).

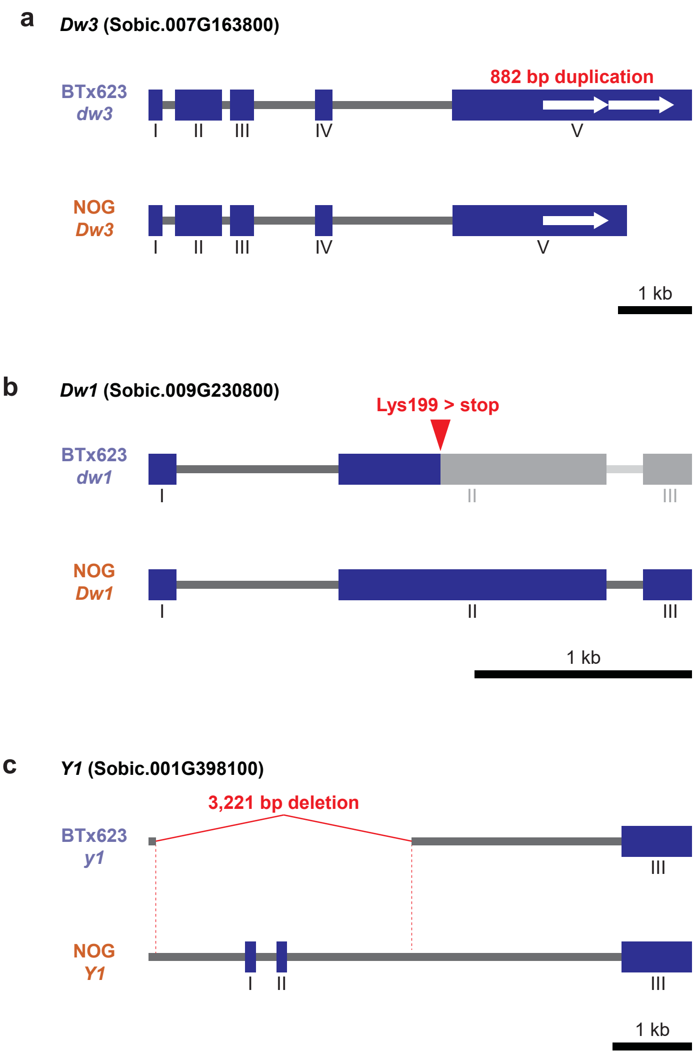

Supplementary Figure S2

The gene structures of *Dw3* (a), *Dw1* (b), and *Y1* (c) in NOG and their mutations in BTx623. Navy boxes represent coding exons. Primers used in study are listed in Supplementary Table S2.

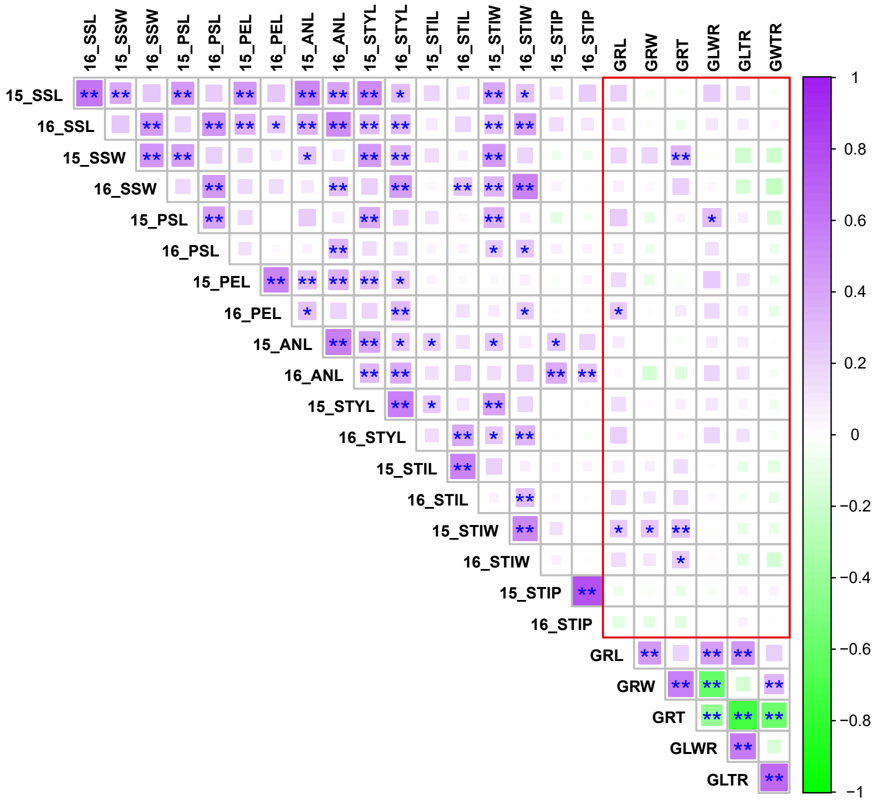

Supplementary Figure S3

Phenotypic correlations for the nine spikelet-related and six grain-related traits measured in this study. Pearson' s correlation coefficients for each trait measured in the RIL population. Purple and green colors represent positive and negative correlations (r) between traits, respectively. SSL; sessile spikelet length, SSW; sessile spikelet width, PSL; pedicellate spikelet length, PEL; pedicel length, ANL; anther length, STYL; style length, STIL; stigma length, STIW; stigma width, STIP; stigma pigmentation, GRL; grain length, GRW; grain width, GRT; grain thickness, GLWR; grain length/width ratio, GLTR; grain length/thickness ratio, and GWTR; grain width/thickness ratio. For the spikelet-related traits, “15_” or “16_” indicate the results of 2015 or 2016. *P < 0.001, **P < 0.0001.

a

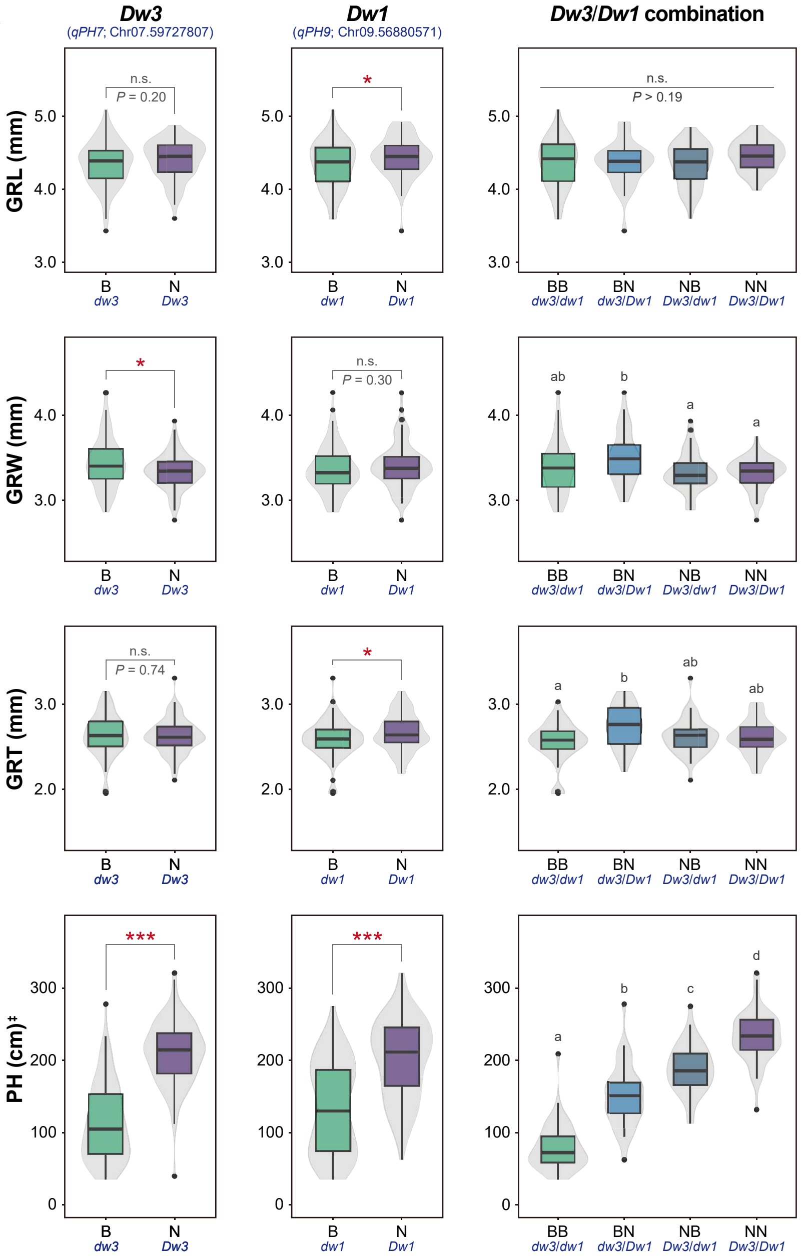

b

	dw3	*Dw3*	Effect of *dw3*†	*dw1*	*Dw1*	Effect of *dw1*†
GRL (mm)	4.35 (0.31)	4.40 (0.26)	1.26% down	4.33 (0.32)	4.45 (0.27)	2.57% down
GRW (mm)	3.44 (0.30)	3.34 (0.20)	3.02% up	3.36 (0.27)	3.40 (0.26)	1.23% down
GRT (mm)	2.64 (0.25)	2.62 (0.20)	0.43% up	2.59 (0.22)	2.67 (0.22)	3.03% down
PH (cm)‡	113.99 (52.48)	210.27 (43.42)	45.79% down	135.35 (62.82)	201.30 (54.92)	32.76% down

Supplementary Figure S4

Relationships between the dwarf genes (*Dw3* and *Dw1*) and grain size in sorghum. (a) Box and violin plots show the effects of the nearest marker genotypes of *Dw3* and *Dw1* to the grain-related traits (GRL; grain length, GRW; grain width, GRT; grain thickness) and plant height. Different letters denote significant differences according to the Tukey-Kramer test ($P < 0.05$). Asterisks indicate significant differences between genotypes (Welch’ s t-test, *$P < 0.05$, ***$P < 0.0005$). (b) The table shows average values and standard deviations (SD, in parentheses) of the population with the mutated allele and that of the functional allele for each dwarf gene. †The ratio of the average value of the population with the mutated allele to that of the functional allele (%). ‡Data of the plant height (PH) were taken from the results obtained by our previous study using the same population (47).

Supplementary Table S1. QTL correspondence with other studies

Chr	QTL cluster	QTLs detected in this study: QTL ID[a]	LOD	PVE (%)	Nearest marker	Marker interval (bp)[b] Start	Marker interval (bp)[b] end	Interval (bp)	Related QTLs reported in other studies: QTL ID[c]	Trait	Population	QTL (bp)[d] Start	QTL (bp)[d] end	Interval (bp)	Reference
1	-	*16_qANL1*	4.34	8.35	Chr01.65134709	64,608,936	65,571,022	962,086	QPLEN1.10	Panicle length	BTx623/IS3620C	64,461,975	66,222,235	1,760,260	Brown et al 2006
									QDMGR1.16	Dry matter growth rate	Diversity set (biomass) (n=194)	65,297,392	65,404,496	107,104	Fiedler et al 2014
		15_qSTIP1	50.62	68.83	Chr01.67767009	67,509,497	68,618,079	1,108,582	QGCOL1.39	Grain color	R890562/ICSV745	67,939,567	68,014,675	75,108	Mace and Jordan 2010
									QGPRA1.19	Proanthocyanidins	Diverse set	68,038,010	68,077,541	39,531	Rhodes et al 2014
		15_qSSW1	6.31	9.81	Chr01.73492838	72,086,062	74,542,240	2,456,178	QFBMS1.6	Fresh biomass	Shihong137/L-Tian	71,558,611	74,007,396	2,448,785	Guan et al 2011
									QGWGT1.21	Grain weight	Sorghum Association Panel (SAP)	73,957,746	74,039,407	81,661	Boyles et al 2016
									QPWTH1.3	Panicle width	BTx623/IS3620C	74,422,759	74,824,330	401,571	Hart et al 2001
		15_qANL1	5.10	9.48	Chr01.78907937	77,228,064	79,222,517	1,994,453	QLFLN1.4	Leaf length	Red Kafir/Takakibi (F2)	77,174,548	80,878,678	3,704,130	Shehzad and Okuno 2015
									QPLEN1.30	Panicle length	Red Kafir/Takakibi (F2)	77,174,548	80,878,678	3,704,130	Shehzad and Okuno 2015
		15_qSTIL1	5.76	9.64	Chr01.80075044	78,907,937	80,451,024	1,543,087	QPWTH1.9	Panicle width	Diverse set	77,687,776	79,617,065	1,929,289	Zhang et al 2015
									QFBMS1.14	Fresh biomass	Shihong137/L-Tian	78,949,693	80,878,678	1,928,985	Wang et al 2016
2	-	*16_qSTYL2*	5.35	8.71	Chr02.67908557	67,181,119	68,205,478	1,024,359	QHGHT2.8	Height (plant height)	Nested Association Mapping set US	66,467,661	67,569,058	1,101,397	Bouchet et al 2017
									QPLEN2.17	Panicle length	Diversity research set (n=107)	68,082,309	68,502,429	420,120	Sakhi et al 2013
3	-	*15_qSTIW3*	9.59	14.51	Chr03.1014391	196,942	1,842,334	1,645,392	QSLEN3.2	Shoot length	Sorghum Association Panel (SAP)	757,282	1,882,419	1,125,137	Chopra et al 2017
									QPLEN3.1	Panicle length	Sorghum Association Panel (SAP)	838,142	1,887,393	1,049,251	Morris et al 2013
		15_qSTIL3	12.71	23.14	Chr03.73116039	72,187,849	73,272,999	1,085,150	QSTHK3.20	Stem circumference	Sorghum Association Panel (SAP)	71,997,455	72,258,061	260,606	Zhao et al 2016
6	-	*15_qPSL6*	6.50	16.87	Chr06.46819000	45,449,430	47,716,228	2,266,798	QHGHT6.48	Height (plant height)	FamilyD: SC1038/(LandracexBTx40	45,391,187	45,542,964	151,777	Higgins et al 2014
									QPLEN6.7	Panicle length	M35-1/B35	46,063,454	46,863,224	799,770	Reddy et al 2013
									QHGHT6.52	Height (plant height)	296B/IS18551	46,449,918	47,615,779	1,165,861	Madhusudhana and Patil 2013
	QC6	*16_qSSW6*	6.59	12.13	Chr06.47921285	47,546,161	48,677,734	1,131,573	QHGHT6.57	Height (plant height)	296B/IS18551	47,028,784	48,266,492	1,237,708	Madhusudhana and Patil 2013
		15_qSTIW6	6.35	9.24	Chr06.48086748	47,740,711	49,157,376	1,416,665	QFBMS6.1	Fresh biomass	Shihong137/L-Tian	47,686,626	50,991,177	3,304,551	Wang et al 2014b
		16_qSTYL6	9.42	16.12	Chr06.48086748	47,921,285	48,677,734	756,449	QPLEN6.12	Panicle length	654/LTR108	48,090,340	50,925,400	2,835,060	Zou et al 2012
		16_qSSL6	3.92	7.09	Chr06.48677734	48,086,748	50,250,487	2,163,739	QHGHT6.88	Height (plant height)	Nested Association Mapping set US	49,724,186	50,568,221	844,035	Bouchet et al 2017
		16_qPSL6	5.34	11.73	Chr06.49336636	48,086,748	50,331,377	2,244,629	QPLEN6.1	Panicle length	Diversity research set (n=107)	49,774,659	50,674,052	899,393	Witt Hmon et al 2014
7	QC7	*15_qPEL7*	11.38	24.21	Chr07.59727807	59,407,685	60,042,098	634,413	QPLEN7.12	Panicle length	296B/IS18551	58,858,415	59,547,856	689,441	Srinivas et al 2009
									QHGHT7.72	Height (plant height)	Shihong137/L-Tian	58,866,126	59,546,987	680,861	Wang et al 2016
		15_qSTYL7	6.69	12.36	Chr07.59727807	59,407,685	60,042,098	634,413	QHGHT7.28	Height (plant height)	BTx623/Rio	59,100,515	59,540,617	440,102	Murray et al 2008
									QHGHT7.75	Height (plant height)	Nested Association Mapping set US	59,529,297	59,532,773	3,476	Bouchet et al 2017
	-	*15_qSSW7*	4.63	7.03	Chr07.64706648	63,878,301	65,440,280	1,561,979	QPLEN7.16	Panicle length	M35-1/B35	63,191,529	65,460,255	2,268,726	Reddy et al 2013
									QGYLD7.3	Grain yield	M35-1/B35	63,221,926	65,460,255	2,238,329	Reddy et al 2013
9	QC9	*16_qSSW9*	6.83	12.62	Chr09.56571248	55,806,970	57,681,027	1,874,057	QHGHT9.30	Height (plant height)	BTx3197/Rio	55,353,960	57,947,320	2,593,360	Felderhoff et al 2012
		16_qSTIW9	7.63	11.69	Chr09.57166357	56,467,290	57,681,027	1,213,737	QHGHT9.19	Height (plant height)	IS8525/R931945-2-2	55,625,467	57,924,294	2,298,827	Wang et al 2014a
		16_qSSL9	8.87	17.03	Chr09.57166357	56,467,290	57,917,278	1,449,988	QHGHT9.11	Height (preflag leaf height	Sorghum Association Panel (SAP)	56,779,950	57,194,940	414,990	Brown et al 2008
		16_qANL9	5.92	11.60	Chr09.57166357	56,571,248	57,917,278	1,346,030	QHGHT9.12	Height (plant height)	ICSV745/R890562-1-2	56,942,211	57,292,395	350,184	Wang et al 2014a
		15_qPEL9	5.12	9.93	Chr09.57599415	56,571,248	58,014,412	1,443,164	QHGHT9.31	Height (plant height)	MS138B/74LH3213	57,091,740	57,916,828	825,088	Takai et al 2012

[a]When overlapped QTLs were detected between years, the one with the smallest marker interval was described as the representative QTL. [b]Marker intervals were estimated based on confidence intervals (2.0-LOD). [c]QTL ID provided by QTL atlas (https://aussorgm.org.au/sorghum-qtl-atlas/). [d]QTL positions estmimated by QTL atlas.

Supplementary Table S2. Primers used in this study

Primer name	Sequence	Comment
Dw1-F	TGTCTTCCCTTCCAGATGCG	Used to amplify entire Dw1
Dw1-R	CTCCTGCGAACGATTGGAGT	
Dw1-SeqF1	CGAGCACGCAAACCCTAACT	Sequencing of Dw1
Dw1-SeqR1	TCTGACAGAAGCAACCAAGCA	
Dw1-SeqF2	CAATCGGGCTTGGGCTTTAG	
Dw1-SeqR2	AGCGTGTGTTAGTCCAAGCA	
Dw1-SeqF3	GGGGCAAAAAGCTTTTACACCT	
Dw1-SeqR3	ATTAGACGTTGGACCGCCAG	
Dw1-SeqF4	CCTGCAGCACGTACTTCTGA	
Dw1-SeqR4	GTCTTCCAATGGGCAGGAGG	
Dw1-SeqF5	TCTGGTGGTTCAGGACTCCA	
Dw1-SeqR5	GCCATCTTTAGCGTAGGCAC	
Dw1-SeqF6	CAGCGGCATCTGCTCAATTC	
Dw1-SeqR6	CTGAGCAAGAATGGCCAGGA	
Dw1-SeqF7	CGCTTGGACGTGTTCAAAGG	
Dw1-SeqR7	CAGCCTACATCATCAGTAAGACCA	
Dw3-F1	GGCTCTCCTGTTCTTCCGTC	Used to amplify entire Dw3
Dw3-R1	TGGTGCAGCGGTTCTACGAG	
Dw3-F2	CTACTTCACCGTCTTCTGCTGCTA	
Dw3-R2	ACTTACAAGGAAGGATCAATTCCCA	
Dw3-F3	ACTACATGGCCACCTTCGTG	
Dw3-R3	CATGCACTACCTTCATTTTTCTGGT	
Dw3-Seq1F	GCCAAGCTCTCCTCCAGGA	Sequencing of Dw3 from exon I to IV
Dw3-Seq1R	CGACGGGTACGCGAAGTC	
Dw3-Seq2F	TCTGTGTGTGTGTGGATCGC	
Dw3-Seq2R	GCTCCAAGCATTCACTTCTTCA	
Dw3-Seq3F	TCGAGAGGTTCTACGACCCC	
Dw3-Seq3R	GCAAAATCGATACGGCAGCA	
Dw3-Seq4F	TGGTCATGCATTCACACAGC	
Dw3-Seq4R	CAGCAGCAGGTTCTCCTTGA	
Dw3-Seq5F	TTGAGCCTTGACCGACTGAC	
Dw3-Seq5R	GCGACGCATGAATGGAGATG	
Dw3-Seq6F	CACGCAGGTCCGTATCGTAT	
Dw3-Seq6R	CCATTCATCTCCCTCTGCCC	
Dw3-Seq7F	AGGGATGGGAGAGCAATTGC	
Dw3-Seq7R	TGTGGCCCATATCCCATGTG	
Dw3-Seq8F	TGATCCCAGTGCAAGCAAGA	
Dw3-Seq8R	AGTACCACTCCTCCAGCATA	
Dw3-Seq9F	TGGTCGAAATTGACGAGACGA	
Dw3-Seq9R	GGATCAGCTTGGCGTAGGTG	
Dw3-Seq10F	TCTTTTGAACCTAGTTAGTCTTTGATT	Sequencing of Dw3 exon V
Dw3-Seq10R	GGTGCCCATCTCGGAGAC	
Dw3-Seq11F	GGGCAGAAGCAGCGCATC	
Dw3-Seq11R	GTAGTAGACGCTGAGCACGG	
Dw3-Seq12F	CCTCTCCGACTTCTCCACCT	
Dw3-Seq12R	ACATCTTCTGCAGGACGGTG	
Dw3-Seq13F	GTGTGCGCGAGAAGATGTTC	
Dw3-Seq13R	CGTTTTCCGGTCGATGGTCT	
Dw3-Seq14F	CAAGATCACGGGGCTCTTCG	
Dw3-Seq14R	ATGATGGCCGCCTGCTTC	
Dw3-Seq15F	GTTCTACGAGCCCACGTCC	
Dw3-Seq15R	CGTTTTCCGGTCGATGGTCT	
Dw3-Seq16F	CTTCTGGAAGGGGCAGATCG	
Dw3-Seq16R	GCAGCGCCGAGATGAACC	
Dw3-Seq17F	GTTCTACGAGCCCACGTCC	
Dw3-Seq17R	GCTTCAGCTTCCTCATCCATC	
Dw3-Seq18F	CTCAAGCACCATCCCGACG	
Dw3-Seq18R	TCTGCCGCTGAAAATCGAGA	
Y1-F1*	ACACACTGCGAGCTGAGAG	Used to amplify Y1 from exon I to exon II
Y1-R1*	GACGTCGGCCCGAAGGTAGTTGATCC	
Y1-F2*	CAAGAACTACTGGAATTCGCACCT	Used to amplify Y1 exon III
Y1-R2*	AGTACAGTACATGTGAAGAAG	
Sobic.004G237000_Fw	GTCCACACACACACACCTTC	Used to amplify and sequence Sobic.004G23700[illegible]
Sobic.004G237000_Rv	AGGAGCTAAAGTGAGGAGGC	
Sobic.004G247000_Fw	AGGTCGAGTAGGGTGTAGGT	Used to amplify and sequence Sobic.004G24700[illegible]
Sobic.004G247000_Rv	CCACCCACTAACCATTCCCT	
Sobic.004G247000_S1	GTTACTACAAACACGTCTTG	Used to sequence Sobic.004G247000
Sobic.004G247000_S2	ACCTAGTTAGTACATGATTG	
Sobic.004G247000_S3	AATAATAATAGGGACCACTC	
Sobic.004G247700_Fw	TCAGCCAAATGTCCAGACCT	Used to amplify and sequence Sobic.004G24770[illegible]
Sobic.004G247700_Rv	CACCCCTGTAGATCGATCGA	
MSD2_Fw	GTCCTCACTTGCTCCCAGTC	Used to amplify and sequence MSD2
MSD2_Rv	ATCGGTACCGCGAGAGACTA	
MSD2_S2	ATTTTACTTACGCCTGGCG	Used to sequence MSD2
MSD2_S3	CTAACATTTCGTCACGCACG	
MSD2_S4	CCACTGCTCGCTCATCTCAC	
MSD2_S5	CAGATCAATCAATCACATGA	

*Y1 alleles for BTx623 and NOG were checked using previously reported primers (Ibraheem *et al*. 2010)
